# hERG Blockade Prediction by Combining Site Identification by Ligand Competitive Saturation and Physicochemical Properties

**DOI:** 10.3390/chemistry4030045

**Published:** 2022-06-21

**Authors:** Himanshu Goel, Wenbo Yu, Alexander D. MacKerell

**Affiliations:** 1 Computer Aided Drug Design Center, Department of Pharmaceutical Sciences, University of Maryland School of Pharmacy, 20 Penn St. Baltimore, MD 21201, United States

**Keywords:** SILCS, hERG channel, Physicochemical properties, Multiple linear regression, FragMaps

## Abstract

Human ether-a-go-go-related gene (hERG) potassium channel is well-known contributor to drug-induced cardiotoxicity and therefore an extremely important target when performing safety assessments of drug candidates. Ligand-based approaches in connection with quantitative structure active relationships (QSAR) analyses have been developed to predict hERG toxicity. Availability of the recent published cryogenic electron microscopy (cryo-EM) structure for the hERG channel opened the prospect for using structure-based simulation and docking approaches for hERG drug liability predictions. In recent time, the idea of combining structure- and ligand-based approaches for modeling hERG drug liability has gained momentum offering improvements in predictability when compared to ligand-based QSAR practices alone. The present article demonstrates uniting the structure-based SILCS (site-identification by ligand competitive saturation) approach in conjunction with physicochemical properties to develop predictive models for hERG blockade. This combination leads to improved model predictability based on Pearson’s R and percent correct (represents rank-ordering of ligands) metric for different validation sets of hERG blockers involving diverse chemical scaffold and wide range of pIC50 values. The inclusion of the SILCS structure-based approach allows determination of the hERG region to which compounds bind and the contribution of different chemical moieties in the compounds to blockade, thereby facilitating the rational ligand design to minimize hERG liability.

## 1. Introduction

The hERG potassium channel plays an extremely important role in heart function through conducting the electrical activity of the heart. Blockade of the hERG channel can lead to QT interval (time measured in electrocardiogram from Q wave to end of T wave) prolongation (long QT syndrome) which can increase the risk of deadly cardiac arrhythmias or torsades de pointes (TdP) leading to sudden death.[1–6] Many drugs (e.g astemizole, cisapride, sertindole, terfenadine etc.) have been withdrawn from the market or their usage restricted due to drug-induced arrhythmias and other side effects.[7–9] Therefore, hERG inhibition by drug candidates has become an important concern in pharmaceutical development.[10,11] Indeed, all potential drug candidates have to be scrutinized against the hERG channel to evaluate their consequence on heartbeat or other cardiovascular side effects as established by the Food and Drug Administration (FDA) and the European Medicine Agency (EMA).[11–15]

Given these complexities, in silico models offer the prospect to effectively eliminate potential drug candidates that could lead to hERG blockade, thereby saving the time and cost of drug development.[16–20] Moreover, apriori prediction of a compounds cardiotoxic potential would be of great utility during the early phase of the drug development process. In this context, a large number of ligand- or knowledge/machine learning (ML)-based drug design models in the context of QSAR have been developed and successfully utilized to rapidly screen drug candidates for their ability to be a hERG binder or nonbinder.[21–26] Pharmacophore[27–30] and structure-based approaches employing homology models[31–34] are another avenue developed to filter drug candidates. A recent comprehensive review of hERG ligand structure activity relationships has been presented.[35] Moreover, the present-day ML based models are getting better for classifying compounds as indicated by improved correlation scores.[36–44] These models largely depend on strong similarity of the candidate drugs to the chemical scaffold and/or different set of properties used to train the models.[45,46] These methods would likely fail to recognize blocker from non-blocker when challenged by a novel compound whose structure or physiochemical properties are not related to the training set of ligands.

The availability of the cryo-EM structure of the hERG channel from Mackinnon and coworkers [47] unlocked the potential to use structure based drug design (SBDD) approaches for hERG screening of drug candidates. Beyond ligand-based methods, SBDD approaches may yield an understanding of the underlying atomistic interactions and binding orientations contributing to hERG binding and blockade. Accordingly, a number of research groups have applied structure-based simulation and docking approaches in modeling ligand interactions with hERG channels, revealing the benefits the SBDD approach can offer when compared to ligand-based techniques.[48–57] A contribution in this direction was recently reported by Mangiatordi and coworkers for a large number of compounds, where they showed the utility of docking scores and their integration with protein-ligand interaction fingerprints.[58]

The Site Identification by Ligand Competitive Saturation (SILCS) technology is unique and innovative among SBDD approaches in that is applies a pre-computed ensemble strategy in a computer-aided drug design framework that produces accuracy similar to state of the art methods such as free energy perturbation while being 100s of times more computationally efficient.[59,60] SILCS methodology involves running a combined oscillating chemical potential grand canonical Monte Carlo[61] and molecular dynamics simulation (GCMC/MD) of small solutes representing different chemical functionalities in the presence of the targeted protein in an explicit aqueous solution.[61–63] This results in generating functional group binding affinity patterns (FragMaps) based on the solute 3D probability distributions which may then be used in the identification of druggable binding sites,[64] for protein-ligand relative binding affinities,[65–68] pharmacophore model development and database screening,[69,70] to evaluate protein-protein interactions,[71] facilitate the identification of excipients for biologics formulation,[72,73] and identify vital contribution of individual atoms and moieties contributing to ligand binding. A recent review article about different SILCS applications[74] can be referred to for additional details.

Taking advantage of the ability of SILCS to predict binding sites and relative affinities, in collaboration with the Noskov research group, the SILCS method was applied to the hERG structure 5VA1 and used to develop a SBDD predictive model of hERG blockade. In that study, which represents the methodological foundation for the present work, SILCS FragMaps were generated for hERG and used to identify binding sites on the channel into which 55 known blockers were docked and the relative affinities determined allowing for correlation analysis with available experiment data. Results from the SILCS approach were compared to results from ligand binding using the GLIDE docking approach.[75] We move beyond that work in the present study by using the SILCS methodology singlehanded and in combination with ligand-based physicochemical properties for predicting the binding affinities and rank-ordering of the hERG blockers/non-blockers for a significantly wider variety of previously published hERG datasets than used in the original study.

As presented below, a major challenge to the development of successful hERG blockade models is a consistent database of hERG compounds with binding affinities measured using a uniform experimental protocol (experimental details, assay type, cell-lines etc.) on a range of chemical scaffolds. To overcome this we have accessed multiple previously published compounds sets curated with different emphases.[24] The training set includes a diverse collection of structural classes of compounds having a wide range in experimental pIC50 values from approximately 2 to 8. The accuracy of the SILCS-based models are then validated in modeling a wide range of hERG compounds coming from different sources.[24,76,77] In addition, we apply a Bayesian ML approach to improve the predictability of the SILCS model as well as combine the structure-based information with physiochemical properties. The resulting SBDD hERG blockade prediction models are equivalent or slightly improved over LBDD ML models while having the benefit of the quantitative evaluation of the binding contributions of individual chemical moieties in the molecules, information available from the SILCS that may be used to predict possible chemical modifications to limit hERG blockade.

## 2. Computational Details

The present study used the SILCS FragMaps calculated in our previous study of hERG blockade model development.[75] Briefly, the SILCS methodology involved running GCMC/MD simulations for the cryo-EM hERG structure (PDB 5VA1)[47] on which the regions not present in the structure had been modelled as previously described,[78] in the presence of water and set of solutes representing different chemical functionalities to generate the functional group affinity patterns (FragMaps) for all the solute molecules. The eight solutes were benzene, propane, methanol, formamide, acetaldehyde, imidazole, methylammonium, and acetate. The simulation system included the hERG channel surrounded in 2-oleoyl-1-palmitoyl-sn-glyecro-3-phosphocholine (POPC) lipids along with cholesterol at a 9:1 ratio. From the SILCS simulations, normalized 3D probability distributions of the functional groups in the solutes encompassing the entire protein are obtained and converted into grid free energy (GFE) FragMaps based on Boltzmann transformation of the normalized probability distributions. The GFE FragMaps, listed in Table S1, Supporting Information, act as the basis for the SILCS-based SBDD. Details of the simulation set up, software, and FragMaps construction may be obtained from our previous hERG publication.[75]

Ligand docking in the GFE FragMaps is performed using the SILCS-Monte Carlo (SILCS-MC) sampling method performed in the field of the GFE FragMaps. Docking was performed individually targeting the previously identified S1 and S2 sites, both of which are located in the intracellular central cavity of hERG.[79] In a recent structure of hERG the blocker astemizole was observed to bind in the vicinity of Y652, which is located in the S1 site. The SILCS-MC docking program involves running energy minimization, MC moves, and MC simulated annealing steps on each ligand in the field of the FragMaps to obtain the lowest ligand grid free energy (LGFE) conformation and orientation of the ligand. LGFE values are the summation of the atomic GFE scores, where the GFE values are computed based on assigning selected atom to a FragMap type and obtaining the atomic GFE scores based on the overlap of the ligand atoms and the corresponding FragMaps. The LGFE values are not formal absolute binding energies but considered as an approximate representation of the experimental binding free energies. The predicted LGFE is converted to pIC50 values for comparison with the experimental values. The ligands were first energy minimized in the context of the CHARMM General Force Field energy function for 10,000 steps followed by 10,000 MC steps at 300 K and 40,000 annealing steps from 300 to 0 K. The Monte Carlo moves include translations, rotations, and dihedral rotations of rotatable bonds that are accepted or rejected based on the Metropolis criteria. The Generic Apolar Scale Atomic Classification Scheme (GAS18 or 2018 Generic ACS) definitions are used for the FragMap classification of solute atoms (Table S1). The force field parameters for the all compounds were obtained from the CGenFF[80–82] program. Prior to SILCS-MC the ligand is randomly placed in the radii of a user specified radius (10 Å in either the S1 or S2 pocket). Five independent simulations of up to 50 MC runs for each ligand were carried out to improve sampling and obtain the minimum energy configuration based on a convergence criteria of 0.5 kcal/mol or a maximum of 250 runs.

Improved predictability of the SILCS model was performed using the previously presented Bayesian machine learning (BML) optimization approach.[67] BML involves using Markov-chain Monte Carlo simulated annealing (MCSA) approach to optimize the weighting factors of the contributions of the FragMaps to the ligand LGFE scores used in the SILCS-MC docking platform. This method allows for the optimization of GFE contributions of the classified atom types from the different FragMap types, representing the features, resulting in maximizing either the Pearson’s correlation (R) or percent correct (PC) metric targeting the experimental binding affinities of the ligands. The BML optimized weights were then used to redock all the compounds in the training and validation sets from which the final R or PC metrics were obtained. In this study, the targeted error function is the Pearson’s R metric for determining new FragMap weights. The force constant for the flat bottom potential was set to 5000 kcal/mol with the lower (0.05) and upper (2.0) limits of FragMap weighting factors. Additional details on the SILCS-MC docking and BML optimization can be referred in our earlier publications.[65,67] Furthermore, a simplified flow diagram has been shown in Figure S1 for depicting the entire computational workflow.

The present study uses multiple sets of diverse ligands curated in previous studies as follows. Training was performed on a set of 163 compounds that includes diverse scaffolds and with standardized experimental pIC50 values collected from different publications as described in our previous hERG publication.[75] The 163 blockers compounds were from the 700 compounds originally reported by Wacker et al.[24] selected based on chemical diversity.[75] For validation purposes, the initial test set of 55 compounds is taken from the work of Kramer et al.[76] where the IC50 values were obtained from the same cell-line and similar experimental conditions. This blocker set contains well known compounds varying from high to low binding affinities. Both the neutral and protonation state of all these compounds were considered and corrections were made in the present study on the protonation states from those used in our previous work[75]. LGFE scores were obtained for both the neutral and charged states of the ionizable compounds. In addition to being used directly in model development the LGFE scores from the two states were used to produce an ionization state weighted LGFE score by using the Henderson-Hasselbalch (HH) equation at pH=7.4 as shown in our earlier publication.[75] Additional test sets of 77, 80, and 32 compounds were from Sinha et al.[77] These data sets were carefully constructed to have compounds with wide ranges of pIC50 values and sufficient number of entries from each range of pIC50 values to avoid biasing from over representation. According to the authors, the patch clamp hERG current inhibition assay in human embryonic kidney (HEK) or Chinese hamster ovary (CHO) cell lines were used to measure the experimental pIC50 values for most of the compounds.[77] Finally, the different test sets compiled by Wacker et al.,[24] comprised of large number of compounds collected from the ChEMBL database, were used for additional model validation. The test sets 1, 3, and 4 contain 100, 155, and 73 compounds, respectively, collected from a large number of published articles. Overall, the collection of blockers in the training and validation sets, with the exception of the 55 blocker set, comes from different articles thereby potentially leading to less consistency between the experimental binding data versus data coming from a single source obtained under similar biological conditions. The lack of consistency in these data sets impacts the ability of the developed models to predict their activity, as described below. The structure for all the blockers in the training and test sets along with their binding affinities are provided in the Supporting Information files.

A set of five physiochemical descriptors, namely partition coefficient (logP), solubility (logS), topological polar surface area (TPSA), molecular weight (MW), and van der Waals volume were used to develop a multiple linear regression (MLR) model to predict activity both alone and in conjunction with the LGFE scores. The descriptor values were obtained from the Molecular Operating Environment (MOE) package (Chemical Computing Group)[83]. While additional descriptors can be included in a MLR based or structure activity relationship model for further improvement of the correlation scores,[84–86] this was not undertaken as the goal of this study is to determine whether using common descriptors could benefit the predictability of the models.

Quantitative statistical evaluation of model predictability used multiple metrics. The metrics are mean unsigned error (MUE) between experimental and predicted pIC50 values, Pearson’s R (R), predictive index (PI)[87] and percent correct (PC) metric. Pearson’s R varies between −1 to 1 denoting quality of the fit and direction of the linear relationship between experimental and predicted values. The PI metric ranges between 1 to −1, where 1 is for the 100% true relative predictions and −1 for 100% false predictions, and 0 for the random predictions, signifying the ability of the method to rank order the ligands based on their affinities. The sum of true positive and true negative comparisons for each series of ligands defines the PC metric. All the PC values are averaged values over individual PC values with each compound in the series taken as the reference ligand. The PC metric is of utility in the context of lead optimization where it can be used to select modifications with the potential to improve the desired target activity, in this case decreased hERG binding.

## 3. Results and Discussion

Building on our previous study, the present work focused on systematically expanding the model optimized based on the 163-compound training set, including taking into account corrections to the ionization state of selected ligands (Supporting information). The developed models are then validated against a wider range of test data. These include more focused data sets with respect to compound types, experimental evaluation method and publications from which they were obtained as well as test sets with more diverse compounds from a significantly larger number of publications. The final section presents the utility of the information content from the SILCS-based model that can be used in the rational design of compounds to minimize hERG liability.

### 3.1. SILCS-MC Docking for Training Set of 163 Compounds

Table 1 presents the statistical analysis based on the SILCS LGFE scores for the 163 blockers in S1 and S2 binding pockets with the standard FragMap weighting factors and the BML optimized factors (Table S1, Supporting Information). The correlation scatter plot between the SILCS predicted pIC50 scores versus the experimental pIC50 values with the BML optimized weighting factors for both the binding pocket S1 and S2 is shown in Figure S2. Based on the LGFE scores alone the predictive capabilities of the model are very low with the R and PI correlation terms approaching zero. However, the MUE values for both the S1 and S2 sites is approximately close to 1 indicating that, while not yielding good correlations, the LGFE scores were giving a representative estimate of the overall binding affinities. Accordingly, the BML optimization operation was performed on the same set of 163 blockers in order to improve the predictability of the model. This approach acts to optimize the weighting of the different FragMap types (e.g. apolar, hydrogen bond donor, hydrogen bond acceptor etc.) to improve the predictability of the model while maintaining the quality of the LGFE scores with respect to their ability to predict the magnitude of the pIC50 values. In this approach the same FragMap weighting factors are applied to all the ligands and, importantly, the final results are based on redocking of the ligands into the reweighted SILCS FragMaps. This final step, along with the validation below, assures that the model is not being overfit. When overfitting is performed the new docked orientations deviate significant from the original docked orientation and the predictability of the model significantly diminishes. As may be seen in Table 1 BML optimization shows substantially improved performance of all the metrics producing R values of 0.453/0.408 as compared to 0.108/0.044 for the S1 and S2 sites, respectively. Similarly, the average PC score of 0.643/0.605 is a substantial improvement over the initial values of 0.524/0.509. As mentioned in the above section, the 163 blockers include different scaffolds and a wide range of experimental pIC50 values taken from numerous publications having different experimental conditions. Therefore, it represents a challenge for any docking or other ranking strategy to predict the ligand binding affinities.

To select the final BML model for application to the remaining validation sets, the BML weighted parameters were obtained by targeting 163 training ligands yielding individual S1 and S2 models and by taking minimum LGFE scores from both the pockets yielding three set of BML optimized reweighted FragMaps. All three sets of reweighted FragMaps were tested against 55 neutral and 42 charged ligands. The best R/PC scores come from the S1 BML parameters when compared to others. Therefore, the BML optimized parameters obtained from the training of 163 blockers in the S1 pocket were used for both the S1 and S2 binding sites in the analysis of the validation test sets presented below. The default and BML optimized FragMap weighting factors are shown in Table S1 of the Supporting Information.

### 3.2. Application of SILCS-MC Predictive Models to Validation Sets

**Figure 1.**
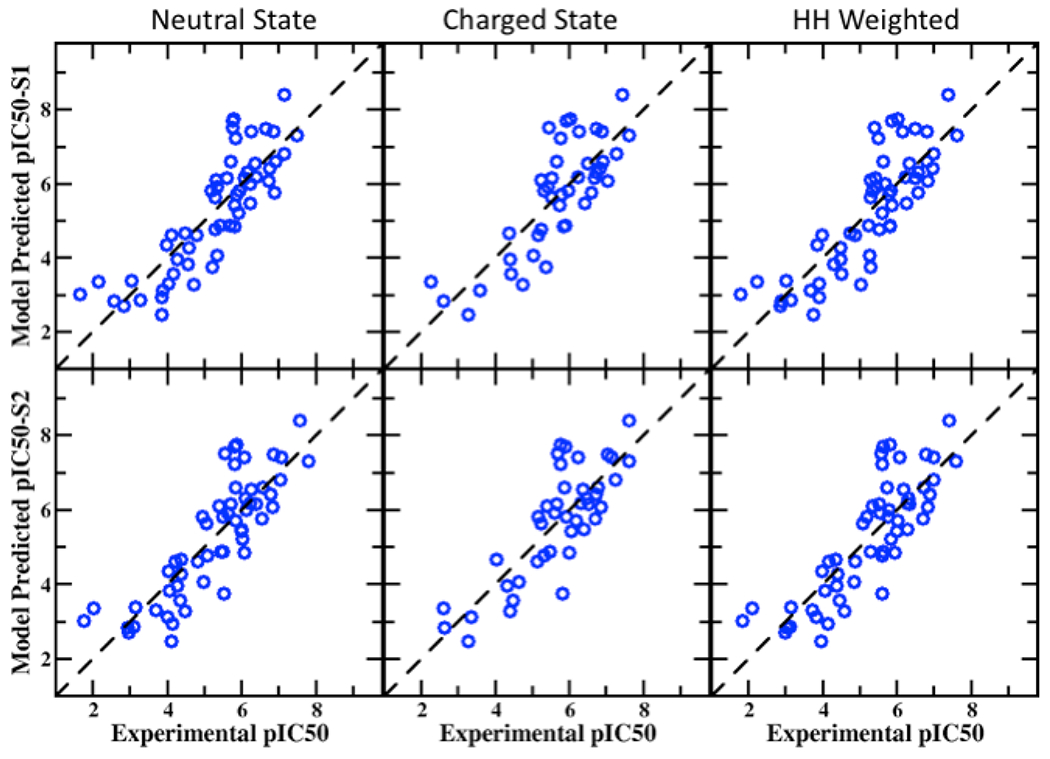
Correlation plots between experimental and predicted pIC50 values from the BML PPM + SILCS LGFE score consensus models for the neutral, charged and HH weighted in the S1(top row) and S2 (bottom row) pocket in the hERG channel.

In this section, multiple ligand sets for validation purposes were used to test the SILCS-MC docking models with and without the BML optimized parameters. The performance of average correlation scores for 55 hERG1 blockers in S1 and S2 binding pocket for neutral, charged and HH-weighted scores are presented in Table 2 with and without BML optimization along with the physiochemical prediction model (PPM) and the combined consensus of PPM and SILCS LGFE scores. A multiple linear regression model was employed to consider the 5 parameters mentioned above. In addition, the three possibilities for treating the charge of the molecules are included in Table 2. The 55 blockers pIC50 values were measured in the same cell-line and similar experimental conditions and contain diverse scaffolds and a wide range of binding affinity values. As may be seen, the predictability of the SILCS models are improved over those optimized directly targeting the 163 compounds in the training set, though the two sets of compounds differ significantly (See Supporting Information Figures S3 and S4 and accompanying text). This emphasizes that using a single set of experimental data benefits to increase the consistency in the pIC50 values and supports the ability of the SILCS computational model to make consistent predictions. The outcomes from the BML optimized model, in particularly for Pearson’s R, is substantially improved in all the states in the S1/S2 pockets when compared to our earlier publication for the same set of blockers after the protonation state corrections performed in this study (see Figure 6 of Mousaei et al.[75]). Furthermore, the performance of the models is sensitive to the ionization state of the compounds with the neutral species yielding a more predictive model than the charges species with the HH weighted model showing the best predictability. Thus, the SILCS BML model indicates the utility of the LGFE metric for predicting hERG blockade. Extending that model with physiochemical properties, yielding the consensus PPM + HH-weighted BML model, clearly shows large improvement as compared to the LGFE scores alone signifying the contributions of the physicochemical properties. However, the PPM only model produces a highly predictive model, approaching that of the consensus PPM-HH-weighted BML model, which is why LBDD hERG prediction approaches are so prevalent in the literature. Comparing the consensus and PPM based model the Pearson’s score with BML S2 pocket for neutral, charged and HH weighted are 0.844, 0.815, and 0.834, whereas the PPM model produces 0.819, 0.784, and 0.809, respectively. Using the physicochemical properties in the consensus model improves the performance of the MUE metric (approximately 0.7 log unit) for all the states in either S1 or S2 pocket. The correlation scatter plot between the SILCS predicted pIC50 scores versus the experimental pIC50 values with BML consensus model are shown in Figure 1 for both the binding pockets S1 and S2. Overall, all the statistical analysis for the standard SILCS-MC and BML optimized models shows considerable improvement in the SILCS model with BML optimization, and using a few physicochemical descriptors further improves the predictability showing the benefits from the combined structure- and ligand-based drug designs approaches.

**Figure 2.**
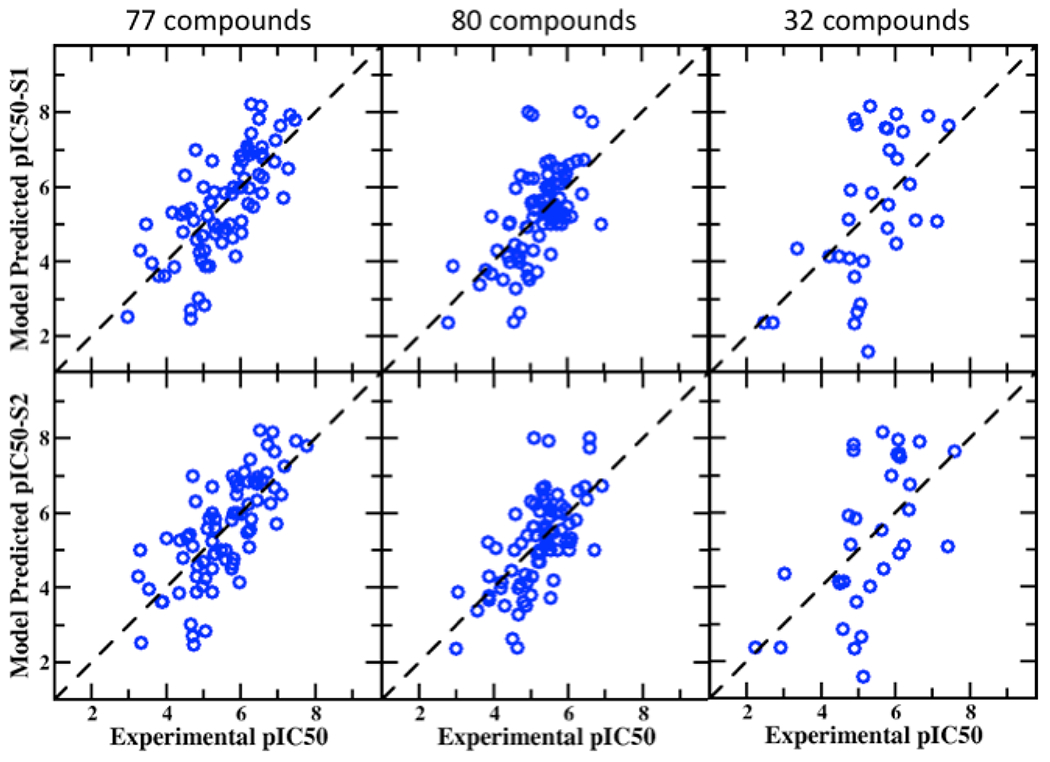
Correlation plots between experimental and predicted pIC50 values from the BML PPM + SILCS LGFE score consensus model for the 77, 80, and 32 compounds in the S1 (top row) and S2 (bottom row) pocket in the hERG channel.

Additional validation sets containing 77, 80, and 32 compounds were obtained from the work of Sinha et al. These have a wide range of experimental pIC50 values from 2.46 to 8.21 and 2.36 to 8.66 and 1.59 to 8.17 for 77, 80, and 32 compounds, respectively. The average correlation scores for these datasets in S1 and S2 binding pocket are presented in Table 3 with standard and BML SILCS models along with the PPM only and consensus PPM/SILCS model. The BML model yields improved performance for the different metrices with all the datasets. The PPM only model again produces quality predictions approaching but slightly worse than the BML/SILCS-based models. The Pearson’s correlation with BML/S2 pocket for 77, 80 and 32 compounds are 0.722, 0.648, and 0.585, whereas the PPM model produces 0.691, 0.516, and 0.540, respectively. Figure 2 provides the scatter plots between the experimental and predicted pIC50 values for visual assessment of BML consensus model based on the S1 and S2 pockets. The MUE accuracy of the BML consensus model for the 77 and 80 dataset varies between 0.71 to 0.78 log unit which is consistent with that of the 55 blockers. However, the 32 compound set yields somewhat larger error close to 1.3 log unit with lower performance of the other statistical metrices as compared to the outcomes from the 77 and 80 compound sets. The 32 compound set also showed somewhat higher error (>1.6 log unit) in the work of Sinha et al.[77] as well. The results from the SILCS and consensus model makes reasonable prediction for the 32 compounds given their chemical diversity.

The final 3 validation sets contain much more diverse ligands collected from a wider range of publications. The performance of different metrices for these validation sets is listed in Table 4 including standard and BML optimized SILCS models along with the solely PPM and consensus PPM/SILCS model with the HH-weighted LGFE scores. The standard SILCS models provides negative or nil correlation for Pearson’s R with all 3 datasets. The BML models makes the predictions somewhat better and provide small positive correlations. Particularly, the prediction from BML S1/S2 test 1 set is better than the other test sets. The consensus PPM/SILCS model further improves the correlation for all the datasets, with the standard and BML consensus models being similar. However, the correlation scores are highly similar to the PPM only model. In other words, considering the binding affinity leads to no further improvement in the model predictability. This appears to be due to inconsistencies in the data as they were extracted from roughly 40, 60 and 80 publications for the 100, 155, and 73 compounds, respectively.[24] In particular, every compound from test set 4 has a unique ChEMBL-assay ID after removal of the duplicates. This illustrates the extreme diversity of compounds in these datasets coming from different experimental procedures, making it difficult to deliver reliable predictions for any predictive model as is seen by the improved prediction metrics in the test sets in Table 2 and 3. Consistent with this, the Wacker et al.[24] article shows poor correlation performance for test sets 3 and 4 with their ML-based platform. The SILCS BML and SILCS/PPM consensus models make better prediction for both these test sets.

### 3.3. Molecular Conformation and Atomic GFE Contribution

The hERG channel can bind a wide range of molecules making it difficult to understand the binding mode of the drug and contribution of different moieties in the compounds to hERG binding. Earlier studies have shown that compounds can bind either parallel or perpendicular to the channel axis and often similar sets of residues play important roles in binding.[88] Mutation experiments confirm the crucial contributions of both polar (T623 and S624) and hydrophobic (Y652 and F656) residues to high affinity blockade.[89–92] However, ligand binding orientations may vary depending upon the functional state of the channel, mutated residues and active/inactive channel conformations.[88] Therefore, understanding the role of different regions of ligands to hERG channel binding is quite challenging making it difficult to rationally modify ligands in order to avoid the hERG liability.

**Figure 3.**
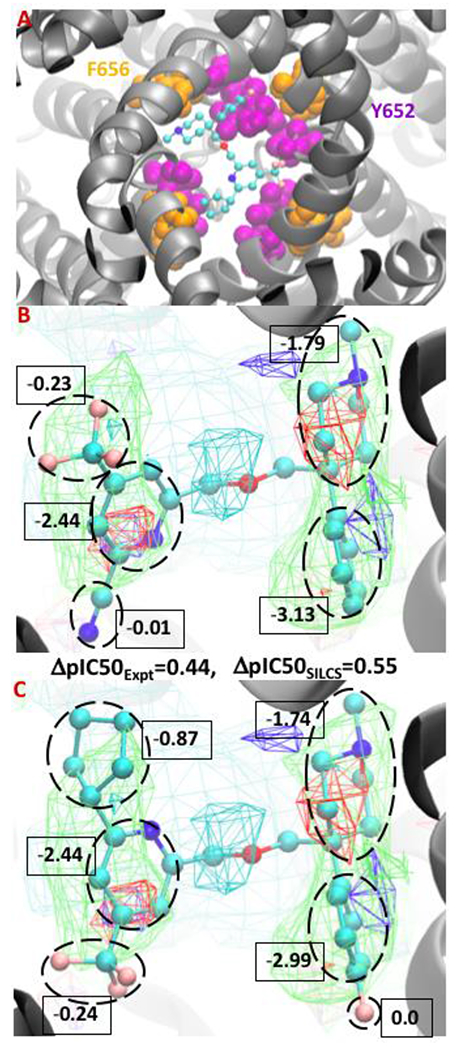
Binding orientation of a compound (taken from Gillman et al.[93]) in the S1 pocket in the vicinity of residues F656 and Y652 (Fig. A). Atomic GFE moiety contributions between the minimum conformation of the congeneric ligands 38 and 51 from test set 1 (Fig. B and C). The FragMaps color are hydrophobic (GENN or APOLAR, green), hydrogen bond acceptor (GENA, red), hydrogen-bond donor (GEND, blue), and positive (MAMN, cyan). The FragMap isocontour surfaces are displayed at a cutoff of −0.8 kcal/mol for the apolar groups and rest of them at −1.0 kcal/mol. The atom cyan, blue, red, and pink, colors represent carbon, nitrogen, oxygen, and fluorine atoms, respectively.

The FragMaps obtained from the SILCS simulations and the ligand conformations from the SILCS-MC docking can reveal the possible molecular interactions occurring between the ligand and protein residues that contribute to binding. This is revealed by the overlap of the SILCS FragMap types with different regions of the docked orientations of the ligands and qualitative understanding of which protein residues contribute to those FragMaps. More quantitatively, the atomic GFE score that are summed to yield the LGFE score allow for the free energy contributions of different regions of the ligands to the binding affinity to be determined. Thus, the SILCS docking approach allows for the identification of crucial atoms and functional group information that allows for the rational design of modifications to minimize binding to the hERG channel. This information can be of great use for lead optimization efforts and understanding how different moieties of a compound contribute to the binding affinity. To exemplify this the binding of two compounds from Gillman et al.[93] are shown in Figure 3. Figure 3A shows the minimum energy conformation of a compound in the region of hydrophobic residues Y652 and F656 indicating the importance of strong hydrophobic interactions. The FragMaps also identify the contribution of the protonated basic nitrogen through positive FragMaps as shown in the center of the Fig. 3B/C which is consistent with the experimental predictions.[94,95] An example in this context is the cationic form of the antihistamine drug astemizole that overlapped with the positive FragMap as shown in our previous work.[75] Moreover, Figure 3B and C presents a ΔpIC50 comparison between the minimum conformation of two congeneric ligands. The hydrophobic group of the compounds occupy the SILCS apolar FragMaps on both sides resulting in favorable GFE contributions those groups.

Comparison of the two ligands shows two modifications on each side of the congeneric molecules. On the right side of the molecule the benzene ring of the ligand was substituted with a fluorobenzene and on the left the cyano group is replaced with a cyclopentyl group. Upon going from the first to second molecule there is a ~180˚ rotation allowing the cyclopentyl group to make favorable contribution due to overlapping with the apolar FragMaps (green) and occupying the hydrophobic pocket along with the connected pyridine ring. Overall, the modifications result in a computed ΔpIC50_SILCS_ score of 0.55 log units, matching well with the ΔpIC50_Expt_ value of 0.44 log units. The GFE contribution of the full cyclopentyl group is −0.87 kcal/mol making the dominate contribution to the change in affinity upon going from compound in 3B to 3C. The GFE contribution of the pyridine ring remains identical in both the ligands as does the trifluoromethyl group despite its change in position in the binding pocket. Interestingly, on the right side, the contribution of the fluorine atom is nil due to not having any acceptor FragMaps in that location and there is no substantial difference in the summed GFE score of the fluorobenzene (−2.99 kcal/mol) versus the benzene ring (−3.13 kcal/mol). As is evident the atomic GFE contributions can be of great use for efficiently designing the ligands with lowered hERG binding, information which is not accessible in other CADD binding affinity methods.

## 4. Conclusions

In the present article, an effective strategy of combining structure- and ligand-based drug design approaches is presented for modeling the relative binding of drug-like molecules to hERG. For this purposes, multiple validation sets were considered and the ability of SILCS methodology was examined independently and in conjunction with simple physicochemical properties through MLR. The predictive model yields sensible performance for binding affinities, Pearson’s correlation, and rank-ordering of the binding (percent correct) for different sets of hERG blockers, especially the ones which come from a single source or based on a consistent experimental set up. Therefore, consistency of the experimental data is very important for SBDD to satisfactorily predict relative affinities. The predictability of the BML-optimized SILCS model shows large improvement in the performance when compared to the outcomes of standard SILCS-MC docking. It’s important to emphasize that the reweighting of the FragMap contributions was performed only for the set of 163 blockers with those weighting factors making satisfactory predictions for different validation datasets. Accordingly, the applicability domain of the developed model may be considered to encompass a wide range of structural classes given the overall predictability of the model for the different validation sets and given the diversity of the compounds in 163 compound training set and the 55 compound validation set as determined form the Jaccard-Tanimoto similarity analysis (Supporting information Tables S3 and S4 and supporting text). Thus, use of the presented model simply requires docking of novel ligands into the presented BML SILCS FragMaps, a process that requires only minutes, along with calculation of the required physiochemical properties. The SILCS software suite is available from SilcsBio LLC and is free of charge to academic users.

Similar to previous SILCS studies, the BML-optimization technique is an effective way to improve the predictability of the model provided experimental information for the training set of ligands is available. Notably, this training set can be small, approaching 10 molecules as shown in our previous studies.[65,67] In addition, the predictability is enhanced with the addition of physiochemical molecular descriptors. Thus, the SILCS SBDD predictive model alone cannot handle hERG compounds obtained from diverse studies. This is consistent with studies showing the need to use specific ML classifier algorithms for handling miscellaneous set of compounds based on the varied binding mode (*e.g*. parallel, perpendicular or alternate orientation), diverse experimental range of pIC50 values, and diverse chemical scaffold as shown in the literature.[19,88] Altogether, the SILCS methodology integrated with few physicochemical properties offers the benefit of predicting relative hERG blockade while offering the advantage of quantitatively understanding how individual moieties in a compound contribute to the change in binding affinity, information that may be used to rationally limit hERG binding.

## Supplementary Material

SI

## Figures and Tables

**Table 1.** Statistical analysis for SILCS models developed based on the 163 hERG1 blockers in the S1 and S2 binding pockets with and without BML optimization.

Pocket	MUE	R	PI	PC
S1	0.836	0.108	0.097	0.524
S2	1.109	0.044	0.034	0.509
BML S1	0.903	0.453	0.456	0.643
BML S2	0.840	0.408	0.362	0.605

**Table 2.** Statistical analysis for 55 hERG1 blockers in S1 and S2 binding pocket for neutral, charged and HH-weighted scores obtained using the models from the SILCS LGFE scores, PPM, and consensus SILCS-PPM ranking.

Docking Scores												

	Neutral State				Charged State				HH Weighted			

Pocket	MUE	R	PI	PC	MUE	R	PI	PC	MUE	R	PI	PC

S1	1.600	0.055	0.098	0.546	1.560	0.057	0.108	0.516	1.494	0.134	0.2	0.573
S2	1.745	0.099	0.114	0.546	1.759	0.092	0.179	0.542	1.682	0.15	0.187	0.573
BML S1	1.050	0.640	0.642	0.734	1.036	0.480	0.380	0.635	0.872	0.705	0.680	0.749
BML S2	1.349	0.611	0.579	0.708	1.329	0.476	0.443	0.653	1.179	0.632	0.619	0.722

PPM + Docking Scores												

S1	0.733	0.819	0.827	0.812	0.764	0.784	0.778	0.785	0.752	0.814	0.820	0.805
S2	0.709	0.825	0.825	0.815	0.743	0.792	0.787	0.794	0.721	0.824	0.828	0.816
BML S1	0.696	0.841	0.852	0.833	0.739	0.804	0.796	0.792	0.702	0.835	0.843	0.822
BML S2	0.669	0.844	0.847	0.826	0.704	0.815	0.763	0.780	0.693	0.834	0.818	0.811

PPM												

MLR	0.734	0.819	0.832	0.816	0.764	0.784	0.781	0.786	0.763	0.809	0.818	0.804

**Table 3.** Statistical analysis for 77, 80, and 32 blockers in S1 and S2 binding pocket using the models obtained from the SILCS LGFE scores, PPM, and consensus SILCS-PPM ranking.

Docking Scores												

	77 compounds				80 compounds				32 compounds			

Pocket	MUE	R	PI	PC	MUE	R	PI	PC	MUE	R	PI	PC

S1	1.298	0.416	0.442	0.641	1.550	−0.078	0.066	0.511	1.888	0.235	0.267	0.589
S2	1.567	0.441	0.456	0.643	1.845	−0.014	0.143	0.549	2.055	0.227	0.297	0.607
BML S1	0.888	0.625	0.647	0.732	0.922	0.516	0.510	0.672	1.720	0.313	0.207	0.560
BML S2	1.185	0.646	0.659	0.738	1.345	0.532	0.541	0.686	1.904	0.363	0.392	0.613

Docking Scores + PPM												

S1	0.824	0.692	0.721	0.757	0.803	0.519	0.565	0.691	1.341	0.566	0.592	0.692
S2	0.822	0.691	0.707	0.749	0.796	0.529	0.575	0.699	1.315	0.562	0.549	0.679
BML S1	0.789	0.713	0.738	0.766	0.724	0.624	0.629	0.722	1.341	0.558	0.539	0.663
BML S2	0.775	0.722	0.745	0.771	0.711	0.648	0.642	0.725	1.320	0.585	0.56	0.681

PPM												

MLR	0.822	0.691	0.707	0.749	0.816	0.516	0.568	0.690	1.382	0.540	0.576	0.679

**Table 4.** Statistical analysis for Test sets 1, 3 and 4 in S1 and S2 binding pocket using the models obtained from the SILCS LGFE scores, PPM, and consensus SILCS-PPM ranking.

Docking Scores												

	Test 1 (100 compounds)				Test 3 (155 compounds)				Test 4 (73 compounds)			

Pocket	MUE	R	PI	PC	MUE	R	PI	PC	MUE	R	PI	PC

S1	1.132	0.034	−0.001	0.503	1.021	−0.125	−0.161	0.464	0.888	−0.088	−0.073	0.476
S2	1.330	−0.019	−0.071	0.489	1.127	−0.156	−0.206	0.442	0.889	−0.159	−0.136	0.445
BML S1	1.008	0.242	0.247	0.608	0.868	0.092	0.105	0.555	1.084	0.061	0.094	0.534
BML S2	1.108	0.236	0.219	0.60	0.897	0.063	0.077	0.537	0.842	0.022	−0.002	0.500

Docking Scores + PPM												

S1	0.886	0.396	0.429	0.643	0.541	0.412	0.475	0.659	0.468	0.454	0.469	0.659
S2	0.937	0.325	0.329	0.595	0.541	0.413	0.474	0.658	0.470	0.444	0.463	0.658
BML S1	0.868	0.419	0.428	0.654	0.542	0.412	0.473	0.659	0.468	0.445	0.447	0.651
BML S2	0.866	0.416	0.43	0.656	0.543	0.412	0.481	0.660	0.468	0.445	0.449	0.651

PPM												

MLR	0.872	0.415	0.442	0.658	0.739	0.542	0.475	0.659	0.469	0.444	0.459	0.656

## Data Availability

Information GitHub access of the SDF files and experimental data etc. https://github.com/mackerell-lab/Herg-Ligands
