# Supplementary material for: hERG Blockade Prediction by Combining Site Identification by Ligand Competitive Saturation and Physicochemical Properties
Source: Chemistry (Basel). Author manuscript; Available in PMC 2023 Sep 1. (PMC9881610; doi:10.3390/chemistry4030045)
Supplement: SI [file NIHMS1860657-supplement-SI.pdf]

Supporting Information

for

hERG Blockade Prediction by Combining Site Identification by Ligand Competitive Saturation and Physicochemical Properties

Himanshu Goel, Wenbo Yu, Alexander D. MacKerell Jr.*

Computer Aided Drug Design Center,
Department of Pharmaceutical Sciences,
University of Maryland School of Pharmacy, 20,
Penn St. Baltimore, MD, 21201, United States

alex@outerbanks.umaryland.edu
Tel: 410-706-7442 Fax: 410-706-5017

Table S1. The atom type classification weighting factors of the SILCS FragMaps for the default 2018 Generic atom classification scheme (ACS) and BML-Optimized. BML optimization was only performed on the FragMap types in the 2018 Generic ACS, which is used for the final model. The additional listed SILCS FragMap types, ACEO, FORN, FORO, MAMC, AALO, AALC, IMIN and IMINH, are not included in the 2018 Generic ACS but are included in the table for completeness.

SILCS Type	Weight Default 2018 ACS	BML-Optimized weights
Benzene C (BENC)	0.167	0.353
Propane C (PRPC)	0.333	0.223
Acetate O (ACEO)	0.5	0.5
Acetate C (ACEC)	1	0.1651
Generic Apolar (GENN)	0.333	0.333
Generic H-bond Donor (GEND)	0.5	0.279
Generic H-bond Acceptor (GENA)	0.333	0.055
Generic heterocycle C (GEHC)	0.333	0.563
Methanol O (MEOO)	1	0.1691
Formamide N (FORN)	1	1
Formamide O (FORO)	1	1
Methylammonium N (MAMN)	1	1.0304
Methylammonium C (MAMC)	1	1
Acetaldehyde O (AALO)	1	1
Acetaldehyde C (AALC)	1	1
Imidazole acceptor N (IMIN)	1	1
Imidazole donor NH (IMINH)	1	1

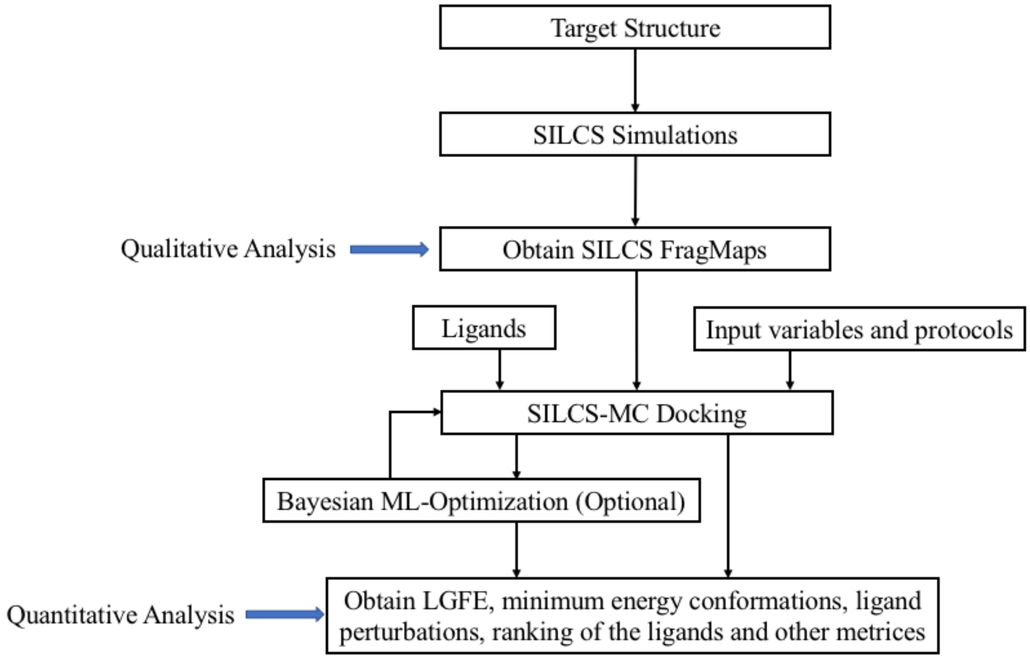

Figure S1: Flow diagram for the entire SILCS workflow.

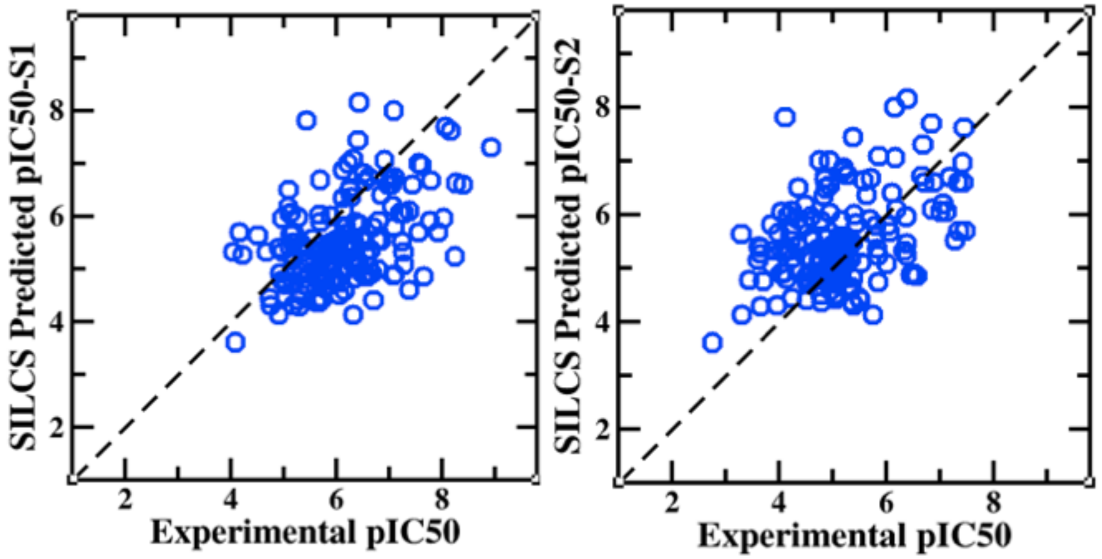

Fig. S2. Correlation plots for the BML SILCS predicted vs experimental pIC50 values for the 163 training ligands in the S1(left) and S2 (right) pocket in the hERG channel.

Chemical similarity of 163 compound training and 55 compound test sets

To quantify the similarity profile of the two data sets, the extended similarity index introduced by Miranda-Quintana et. al. [1,2] was evaluated for both sets. According to their tests on 19 compound libraries [3], the extended Jaccard-Tanimoto (JT) similarity index combined with RDKit fingerprint performed the best. Thus, here we calculated the nonweighted extended JT similarity index with RDKit fingerprints for both sets to quantify their similarity profiles using the python libraries provide by the authors on GitHub [1]. The RDKit fingerprints were calculated using the RDKit library for all molecules. JT similarity index was calculated at no and 9 different coincidence thresholds. As shown in Figure S2, both sets are quite chemically diverse as indicted by the small JT similarity values across different coincidence thresholds. Such similarity profiles are very similar to the Approved Drugs data set tested previously [3].

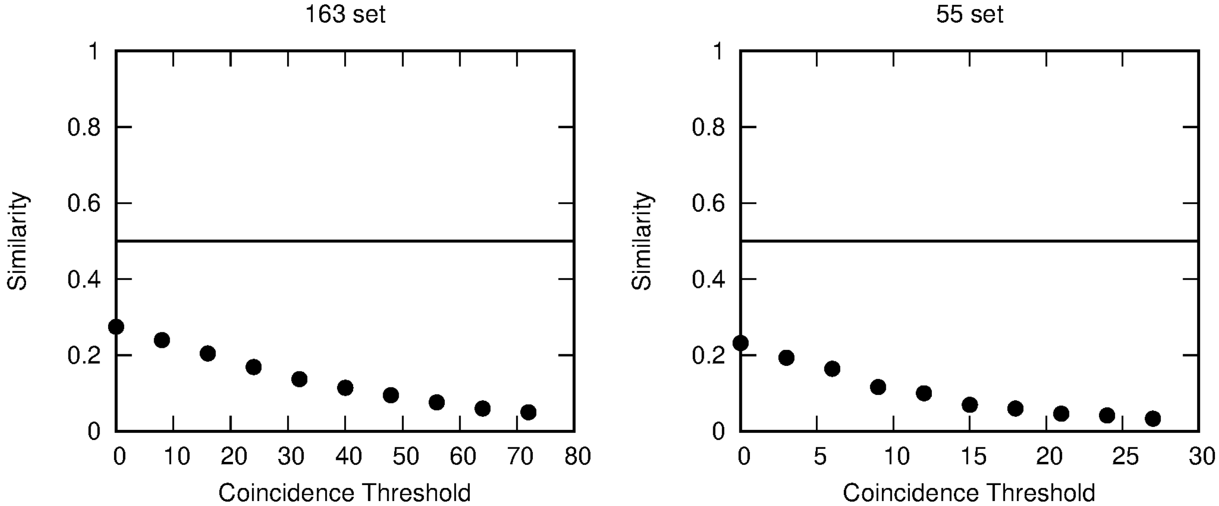

Figure S3. Extended Jaccard-Tanimoto (JT) similarity index versus coincidence threshold for the 163 molecules set (left) and 55 molecules set (right) using the RDKit fingerprint. The 0.5 line is included as reference.

To compare the diversity between the two sets, the suggested absolute and relative diversity [3] were calculated as shown in Figure S3. Both absolute and relative diversity are calculated to be under 0.5 for all coincidence thresholds indicating that the 55 molecule set is slightly more diverse than the 163 molecule set.

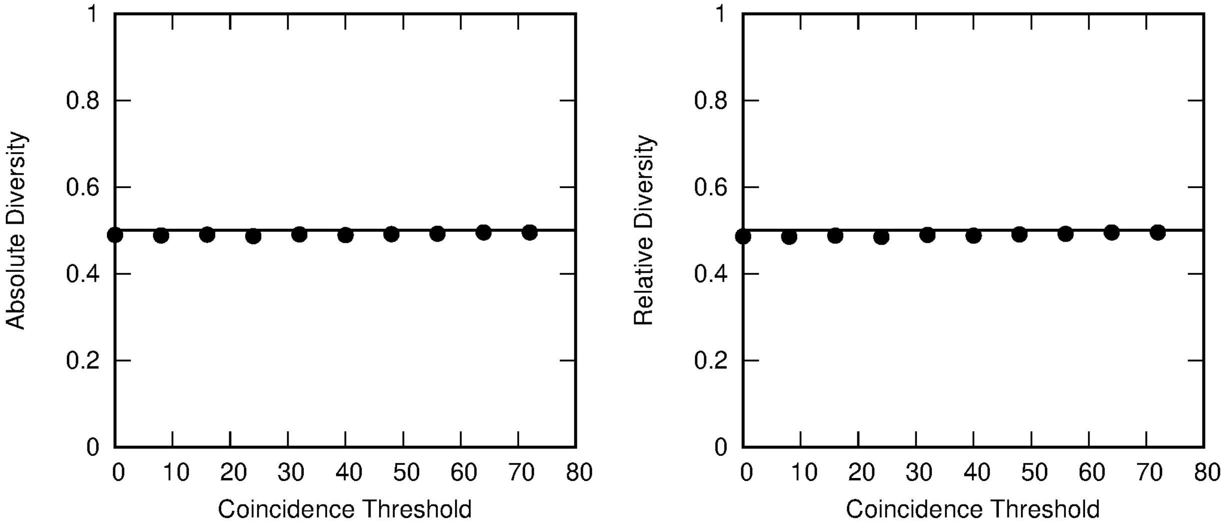

Figure S4. Diversity versus coincidence threshold for the 163 molecules set with the RDKit fingerprint using absolute diversity (left) and relative diversity (right) with respect to the 55 molecules set. The 0.5 line is included as reference.

References

[1] Miranda-Quintana RA, Bajusz D, Rácz A, Héberger K. Extended similarity indices: the benefits of comparing more than two objects simultaneously. Part 1: Theory and characteristics. J. Cheminformatics 2021; 13:32.
[2] Miranda-Quintana RA, Rácz A, Bajusz D, Héberger K. Extended similarity indices: the benefits of comparing more than two objects simultaneously. Part 2: speed, consistency, diversity selection. J. Cheminformatics 2021; 13:33.
[3] Dunn TB, Seabra GM, Kim TD, Juárez-Mercado KE, Li C, Medina-Franco JL, Miranda-Quintana RA. Diversity and Chemical Library Networks of Large Data Sets. J. Chem. Inf. Model. 2021, to be published. https://doi.org/10.1021/acs.jcim.1c01013
